# Isolation and Characterization in a Soil Conditioned With Foaming Agents of a Bacterial Consortium Able to Degrade Sodium Lauryl Ether Sulfate

**DOI:** 10.3389/fmicb.2020.01542

**Published:** 2020-07-07

**Authors:** Ludovica Rolando, Paola Grenni, Jasmin Rauseo, Tanita Pescatore, Luisa Patrolecco, Gian Luigi Garbini, Andrea Visca, Anna Barra Caracciolo

**Affiliations:** ^1^Water Research Institute – National Research Council (IRSA-CNR), Monterotondo, Italy; ^2^Department of Ecological and Biological Sciences, Tuscia University, Viterbo, Italy; ^3^Institute of Polar Sciences – National Research Council (ISP-CNR), Monterotondo, Italy

**Keywords:** anionic surfactant, foaming agents, biodegradation, tunneling, spoil material, bioaugmentation

## Abstract

The anionic surfactant Sodium Lauryl Ether Sulfate (SLES) is the principal component of several commercial foaming products for soil conditioning in the tunneling industry. Huge amounts of spoil material are produced during the excavation process and the presence of SLES can affect its re-use as a by-product. Anionic surfactants can be a risk for ecosystems if occurring in the environment at toxic concentrations. SLES biodegradability is a key issue if the excavated soil is to be reused. The aim of this study was to identify bacteria able to degrade SLES, so that it could potentially be used in bioaugmentation techniques. Enrichment cultures were performed using bacterial populations from spoil material collected in a tunnel construction site as the inoculum. A bacterial consortium able to grow in a few hours with SLES concentrations from 125 mg/L to 2 g/L was selected and then identified by Next Generation Sequencing analysis. Most of bacteria identified belonged to Gamma-*Proteobacteria* (99%) and *Pseudomona*s (ca 90%) was the predominant genus. The bacterial consortium was able to degrade 94% of an initial SLES concentration of 250 mg/L in 9 h. A predictive functional analysis using the PICRUSt2 software showed the presence of esterase enzymes, responsible for SLES degradation. The bacterial consortium selected could be useful for its possible seeding (bioaugmentation) on spoil material from tunneling excavation.

## Introduction

Due to the many and ever larger tunneling projects planned in Europe and worldwide, several hundreds of millions of tons of soil debris (spoil material) will be produced in the coming years, bringing the associated issue of how to manage and dispose of them (Milligan, 2000; Tokgöz, 2013, 2020; Oggeri et al., 2014). In fact, it is estimated that a tunnel with a length of 50 Km and a cross section of 100 m^2^ leads to 8 million m^3^ of spoil material composed of different sizes of broken rocks or soil. The anionic surfactant sodium lauryl ether sulfate (SLES) is the main component in several foaming agents (FAs) used in the excavation of highway and railway tunnels with Earth Pressure Balance-Tunnel Boring Machines (EPB-TBM), (Barra Caracciolo et al., 2017). Foaming agents are used for their ability to modify the mechanic and hydraulic behavior of soil, resulting in a malleable material that can be efficiently excavated and transported to temporary deposit areas (Peila, 2014; Peila et al., 2016). The spoil material could therefore contain the foaming agents used for soil conditioning. The amount of each FA employed, in terms of soil treatment ratios (TR), depends on the specific lithological characteristics and can vary from 0.1 to 3 L/m^3^. The percentages of SLES can range from 5 to 50% depending on the specific commercial product (Barra Caracciolo et al., 2017). A recent study reports concentrations of SLES in various conditioned soils from excavated tunnels in a range of 27–350 mg/kg soil (Finizio et al., 2020). The presence of SLES can influence spoil material reuse as a by-product if its concentrations are toxic for terrestrial and aquatic organisms (Grenni et al., 2018; Galli et al., 2019; Finizio et al., 2020). Soil debris can be used as a by-product for different purposes, such as refilling old quarries and road construction, raw material for industrial production, filling green areas and in some cases beach nourishment. Although at the FA treatment ratios generally used in tunneling, SLES residues in the spoil material are not toxic for terrestrial organisms, they can be potentially hazardous for aquatic ones because the latter are very sensitive to surfactant residues (Grenni et al., 2018; Galli et al., 2019; Finizio et al., 2020). This means that spoil material can have different concentrations of SLES residues depending on the final destination site (e.g., industrial sites or green areas) and on whether or not there will be a contact with water bodies, (Mininni et al., 2018; Grenni et al., 2019; Finizio et al., 2020; Pescatore et al., 2020). No clear framework has been defined by national and international environmental agencies for a chemical and ecotoxicological characterization of excavated material aimed at evaluating its potential environmental effects so far (Mariani et al., 2020). In fact, the spoil material can be a safe by-product if it is temporarily stored at the construction site for the time needed for its biodegradation (Barra Caracciolo et al., 2019). This practice is currently used in several tunneling sites in Italy where SLES is generally found to degrade in about 7 days with an initial concentration of about 200 mg/kg (Mariani et al., 2020). However, SLES degradation rate depends on its overall initial concentration, microbial numbers, soil texture, depth, humidity, temperature, and organic carbon, which are site-specific and can vary in different seasons. Consequently, the degradation of SLES can sometimes require several weeks (Finizio et al., 2020), with a significant increase in the duration of the temporary storage at the construction site. In some cases, such as tunneling for a metro in a city, an area for temporary storage of the spoil material is not available. This forces considering this material as waste, with a significant increase in work costs and soil consumption (landfill). Reducing waste production and re-using by-products are primary objectives of the so-called “circular economy” and in this context the biodegradation of SLES becomes a key point. A possible nature-based solution (Keesstra et al., 2018) for preventing loss of soil and useless waste production can be spoil material bioaugmentation with a bacterial consortium able to promote a quick degradation of SLES.

Sodium lauryl ether sulfate is formed from a predominantly linear aliphatic hydrocarbon chain (length between C_8_ and C_18_) with a polar sulfate or sulfonate group, neutralized with a counter ion (e.g., Na^+^, K^+^, NH_4_^+^, or alkanolamine cation), (Cserháti et al., 2002; Wibbertmann et al., 2011). The best known SLES degradation pathway starts with an initial ether cleavage based on where the initial cut occurs. The resulting intermediate metabolites are polyethylene glycol (PEG) sulfates, which can be additionally degraded by releasing the sulfate ion (Hales et al., 1986; Budnik et al., 2016). Another possible mechanism is ester cleavage, which directly causes the split of the sulfate, before the degradation of the carbon chain (Hales et al., 1986). SLES is a biodegradable compound and some microbial consortia (a group of two or more different species working together) able to degrade it have been isolated from wastewater in laboratory cultures, such as the consortium comprising the genera *Pseudomonas* and *Aeromonas* (Paulo et al., 2017) and the consortium comprising the genera *Serratia, Enterobacter*, and *Alcaligenes* (Fedeila et al., 2018).

In this context, the purpose of this study was the identification and characterization of a bacterial consortium able to degrade SLES in spoil material. Soil samples were collected from a real tunnel construction site and enrichment cultures with different SLES concentrations were performed. The bacterial consortium selected and identified by FISH and NGS techniques in this work is proposed for its potential application in bioaugmentation. Bioaugmentation is a low-cost, environmentally friendly and easily applied solution for achieving rapid SLES degradation and safe re-use of spoil material in tunneling excavation.

## Materials and Methods

### Chemicals

Chloroform of HPLC-grade, hydrochloric acid (37%), sulfuric acid (98%) and methylene blue were obtained from VWR (Radnor, PA, United States). Sodium hydrogen carbonate and anhydrous sodium carbonate were bought from Carlo Erba reagents (Milan, Italy). Water was purified (18 MΩ/cm quality) using a Milli-Q system Millipore (Bedford, MA, United States). SLES (technical grade purity) was purchased from BOC Sciences (United States, Canada). Two SLES stock solutions were prepared at 10 g/L in water and 1 g/L in methanol and stored at 4 and −20°C, respectively. The aqueous solution was used for setting-up the enrichment culture and SLES degradation experiments, while the SLES stock solution in methanol was used for the chemical determinations.

### Enrichment Culture Using Spoil Material as Inoculum

Enrichment cultures were performed using spoil material a railway tunnel construction site in Southern Italy as the inoculum. The soil samples, mainly clayey, were collected at 10–20 m depth. Aliquots of soil (0.5 g in each flask) were added to 50 mL of minimal medium (MB1) enriched with four different SLES concentrations (25, 50, 100, 200 mg/L). The minimal medium (MB1) had the following composition (per liter): 0.8 g K_2_HPO_4_, 0.2 g KH_2_PO_4_, 0.05 g CaSO_4_**.**2H_2_O, 0.5 g MgSO_4_**.**7H_2_O, 0.01 g FeSO_4_, 1 g (NH_4_)_2_SO_4_. The pH of the medium was adjusted to 7.0 before autoclaving. SLES was added as the sole carbon source at different concentrations (25, 50, 100, and 200 mg/L). Appropriate non-inoculated sterile controls were also set up throughout the experiment. Erlenmeyer flasks (three replicates for each concentration) were incubated at 28°C in the dark and maintained on a rotary shaker at 130 rpm for 7 days. The pre-grown cultures from each concentration were then transferred to a new fresh media for another 7 days. This step was repeated for at least four times. The bacterial growth was measured in the form of optical density (O.D.) at 0, 3, 6, 24, 48, 72, 144, and 156 h, using a spectrophotometer (Multiskan Sky Microplate Spectrophotometer, Thermo Scientific) at a wavelength of 600 nm. The culture that showed the highest growth rate (200 mg/L) was tested again at 14 different SLES concentrations (0.5, 1, 2, 4, 8, 16, 31, 62.5, 125, 250, 500 mg/L, 1, 2, and 4 g/L). The bacterial consortium was incubated in a 96 well plate at 37°C with background shaking at 180 rpm. The bacterial O.D. was measured for each concentration tested every 15 min for 24 h, using the Multiskan Sky Microplate Spectrophotometer. Finally, the culture that showed the highest growth rate was used for the isolation of a pure culture and the subsequent SLES degradation experiment.

#### Bacterial Isolation and FISH Analysis

A loopful of the above liquid culture was streaked onto agar plates (MB1 + SLES as sole carbon source) to isolate any pure culture. Different concentrations of SLES were tested (125, 250, and 500 mg/L) for this purpose. The plates were incubated for 3 days at 28°C. Once the bacterial colonies had grown, they were firstly observed under a phase contrast microscopy (1000 ×; Leica 4000B, Leica Microsystems GmbH, Wetzlar, Germany) in order to study their morphology. The phylogenetic composition was then analyzed with the Fluorescent *in situ* Hybridization (FISH) technique (Barra Caracciolo et al., 2005c). For the latter purpose, a single colony was suspended in 2 mL of MB1, fixed with formaldehyde (2% final concentration) and then 100 μL were filtered through a 25 mm white polycarbonate membrane with a porosity of 0.2 μm (Merck Millipore) using a gentle vacuum (<0.2 bar). The filters were treated with oligonucleotide fluorescent probes for the identification of the main classes in the Bacteria domain (e.g., Alpha, Beta, Gamma, Epsilon-*Proteobacteria*) as described in detail elsewhere (Barra Caracciolo et al., 2005b, 2010).

### SLES Biodegradation Experiment With the Selected Bacterial Inoculum

A biodegradation experiment was carried out using the bacterial consortium (ca. 1 mL, BC) selected from the enrichment experiment as the inoculum. The inoculum was added to 50 mL of MB1 containing SLES as the sole carbon source at a concentration of 250 mg/L (MB1 + SLES + BC). Moreover, a chemical control (sterile MB1 with only SLES at 250 mg/L = MB1 + SLES), a microbiological control (Positive control = MB1 + BC) and a blank (Negative control = MB1) were also performed. The flasks (3 replicates for each condition) were incubated at 28°C in the dark and maintained on a rotary shaker at 130 rpm. Cell growth (optical density), bacterial abundance (No. cells/mL measured by DAPI counts), cell viability (% live cells/live + dead, measured by cell viability) and SLES concentration (% residual SLES concentration) were measured at 0, 5, 8, 9, and 24 h. Finally, DNA from cells was extracted to identify the bacterial consortium by Next Generation Sequencing (NGS) from the inoculum and at 8 and 24 h. DNA from the blank at 0, 8, and 24 h was also extracted and sequenced in order to avoid any false positives.

#### Total Bacterial Abundance and Cell Viability by Epifluorescence Direct Methods

Total bacterial abundance and cell viability were observed and counted with a fluorescence microscope (Leica DM 4000B, Leica Microsystems GmbH, Wetzlar, Germany). Briefly, total bacterial abundance (No. cells/mL) was assessed (three sub-sample replicates) using aliquots of samples (ranging from 0.01 to 1 mL), fixed with formaldehyde (2% final concentration), and filtered through a 25 mm black polycarbonate membrane with a porosity of 0.2 μm (Merck Millipore) using a gentle vacuum (<0.2 bar). The filters were treated with DAPI (4’,6-diamidino-2-phenylindole) as described in detail elsewhere (Barra Caracciolo et al., 2005a, b). The total bacterial cell count with DAPI detects the cells in a sample no matter what their physiological and metabolic state.

Cell viability (% live cells/live + dead) was assessed (three sub-sample replicates) using aliquots of fresh samples (the same volumes used for total bacterial abundance) which were filtered through the same filters for DAPI counts. The filters were treated with two fluorescent dyes, SYBR Green II and propidium iodide, as described in detail elsewhere (Grenni et al., 2009). This method can detect microorganism viability because the propidium iodide dye can only enter cells that are dead or with the cellular membrane damaged, and they appear red under a fluorescence microscope. Finally, the live cell abundance (No. live cells/mL) was calculated from the total bacterial abundance (obtained by DAPI counts) multiplied by the cell viability.

#### DNA Extraction, Next Generation Sequencing (NGS) and Predictive Functional Gene Analysis of the Bacterial Consortium

DNA was extracted from the inoculum, from the condition MB1 + SLES + BC at 8 and 24 h and from the blank (MB1) at 0, 8, and 24 h of the SLES degradation experiment. The extraction was performed using the DNeasy PowerSoil kit (QIAGEN), in line with the manufacturer’s protocol, and then was quantified with the Multiskan Sky Microplate Spectrophotometer. The extract quality was checked using the 260/280 and 260/230 nm ratio and 0.8% agarose gel electrophoresis. The genome of the bacterial consortium was sequenced with a next-generation sequencing (NGS) approach, using the MiSeq platform (Illumina) at BioFab Research (Rome, Italy). The V_3_-V_4_ region of the ribosomal gene 16S (16S rRNA) was amplified. The primers used were 341F (CCTACGGGAGGCAGCAG) and 805R (GACTACHVGGGTATCTAATCC), (Klindworth et al., 2013). Raw reads were imported and demultiplexed using QIIME 2 v2019.1^1^ and denoised with the DADA2 plugin (Callahan et al., 2016). The amplicon sequencing variants (ASVs) with less than 0.005% of high-quality reads were then filtered in accordance with Bokulich et al., 2013 to avoid an overestimation of the diversity (Bokulich et al., 2013). The filtered ASVs were compared to the 97% identical clustered Silva database [version 132^2^, (Quast et al., 2013)] using a naive Bayes classifier trained on the amplified region with an 80% reliability. The number of sequences of each sample was rarefied to the lowest one to avoid any bias.

The PICRUSt2 software tool was used to predict functional gene abundances based on the 16S rRNA (Douglas et al., 2019). The ASVs table previously generated was used as the input. The prediction of enzyme commission (EC) relative abundances was performed with the hidden-state prediction (Louca and Doebeli, 2018) and used for inferring pathway abundances (Ye and Doak, 2009).

#### Analytical Determination of SLES

Sodium lauryl ether sulphfate concentration was measured at different sampling times (0, 5, 8, 9, and 24 h) of the SLES degradation experiment following the optimized MBAS spectrophotometric method (Methylene Blue Active Substances). Briefly, the method was based on three consecutive chloroform extractions of the ionic-pair reaction between SLES and the methylene blue; the absorbance of the SLES-MBAS-chloroform complex obtained from liquid-liquid extraction, was measured using a UV/Vis spectrophotometer (650 nm, Perkin Elmer 25). Further details are reported in Barra Caracciolo et al. (2019). Finally, the SLES concentration was obtained using the equations resulting from the standard calibration curves (0.05–4 mg/L SLES) previously determined. The limit of detection (LOD), was 0.013 mg/L (IUPAC, 1999).

## Results

### Enrichment Cultures Using Spoil Material as Inoculum

**FIGURE 1:**
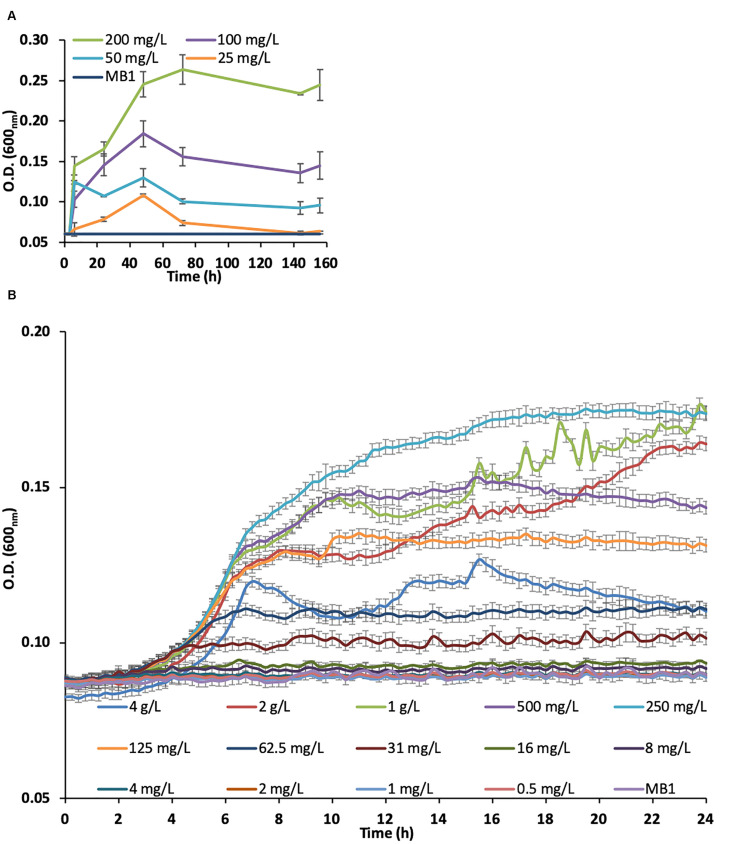
Growth curves of the bacterial consortium on SLES enrichment cultures measured by optical density (O.D.) at 600 nm. **(A)** Enrichment cultures at 4 SLES concentrations (25, 50, 100, and 200 mg/L). **(B)** Bacterial consortium, from the enrichment cultures at 200 mg/L, tested at 14 SLES concentrations. The vertical bars are the standard errors. MB1 = negative control.

A bacterial consortium was selected using enrichment cultures with spoil material as the inoculum. The growth curves of the bacterial consortium in the enrichment cultures using 4 SLES different concentrations (25, 50, 100, and 200 mg/L) are reported in Figure 1A. The bacterial consortium which showed the highest growth rate (m_max_ = 0.064/h) was enriched with 200 mg/L of SLES (Table 1) and tested again with 14 SLES concentrations (from 0.5 mg/L to 4 g/L, Figure 1B). The highest growth rate (m_max_ = 0.028/h) was observed at 24 h with 250 mg/L of SLES (Table 2).

**TABLE 1:** Growth rate of the enrichment cultures at 4 different SLES concentrations.

SLES concentration (mg/L)	200	100	50	25
μ_max_ (h^–1^)^a^	0.064	0.039	0.053	0.007

*^a^μ_max_ = Growth rate during the exponential phase.*

**TABLE 2:** Growth rate of the bacterial consortium grown at 14 different SLES concentrations.

SLES concentration (mg/L)	4000	2000	1000	500	250	125	62.5	31	16	8	4	2	1	0.5
μ_max_ (h^–1^)^a^	0.023	0.023	0.026	0.026	0.028	0.022	0.015	0.011	0.006	0.005	0.003	0.003	0.003	0.002

*^a^μ_max_ = Growth rate during the exponential phase.*

#### Bacterial Isolation and FISH Analysis

The growth of bacterial colonies on Petri plates was only found with 500 mg/L of SLES. The observation of the colony morphology, under a phase contrast microscopy, showed different bacterial shapes. Moreover, the Fluorescent *in situ* Hybridization analysis of the isolates showed that most bacteria identified belonged to Gamma-*Proteobacteria* (88%). The results of the other classes searched for with FISH probes are not reported because no positive fluorescent signals to the other probes were found.

### SLES Biodegradation Experiment

**FIGURE 2:**
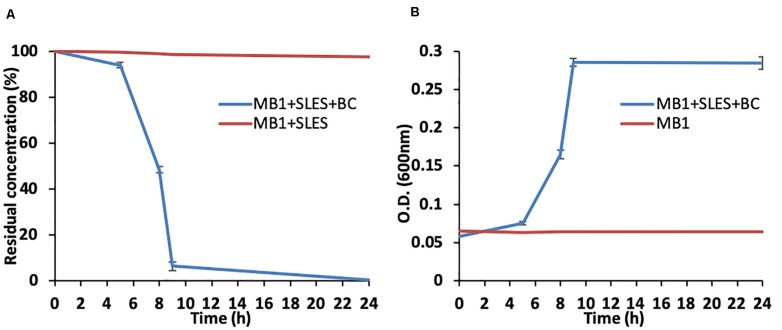
SLES biodegradation experiment. **(A)** SLES decrease due to the bacterial consortium over time (0–24 h). **(B)** Bacterial consortium growth measured as O.D. (600_nm_). The vertical bars are the standard errors. MB1 = negative control.

The SLES degradation experiment showed that 94% of the initial concentration of SLES (250 mg/L) was biodegraded in only 9 h (Figure 2A), when the bacterial consortium reached the maximum concentration (O.D._600__nm_ ∼0.3, Figure 2B). No cell growth and SLES degradation were observed in the chemical control (sterile MB1 with only SLES at 250 mg/L), where the bacterial consortium was absent.

**FIGURE 3:**
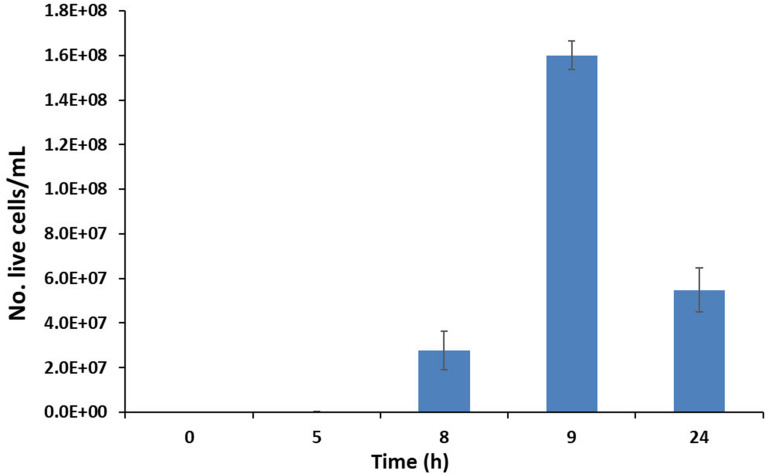
Number of live cells (No. live cells/mL) evaluated using direct fluorescence methods at different experimental times (0–24 h). The vertical bars represent the standard errors.

At the same time as measuring O.D., the bacterial growth was also evaluated using epifluorescence direct methods. The number of live cells (No. live cells/mL) increased significantly over time (Figure 3), reaching its maximum value (∼1.6 × 10^8^) at 9 h, in line with the SLES degradation (Figures 2A,B). A decrease in the number of live cells was observed at 24 h.

#### Next Generation Sequencing (NGS) and Predictive Functional Gene Analysis of the Bacterial Consortium

The NGS analysis of the bacterial inoculum showed a prevalence of Gamma-*Proteobacteria* (>99%). The predominant genus was *Pseudomonas* (92%) and other genera found were *Acinetobacter*, *Stenotrophomonas*, *Pseudoxanthomonas, Cupriavidus* (Beta-*Proteobacteria*) and *Ensifer* (Alpha-*Proteobacteria)*.

**FIGURE 4:**
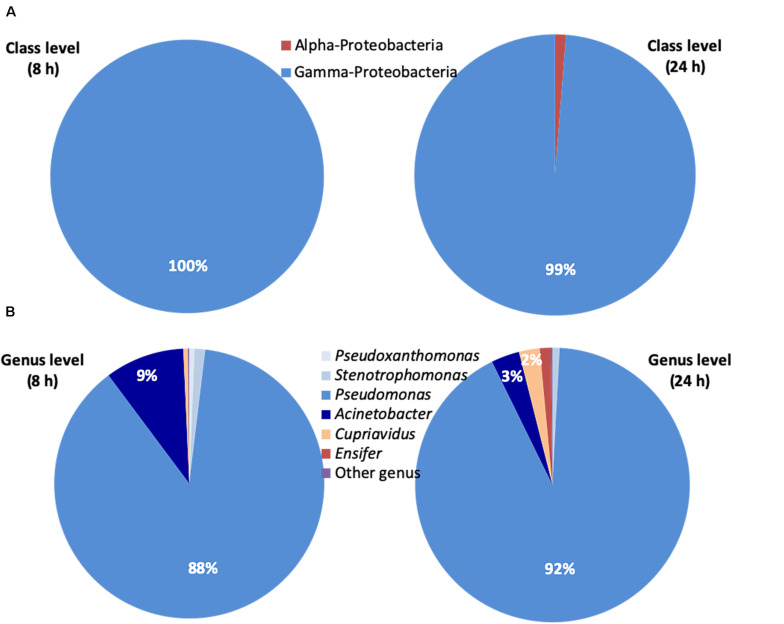
Next Generation Sequencing (NGS) of the bacterial consortium at 8 and 24 h. **(A)** Relative abundance (% of total ASVs) at class level of ASVs belonging to the Bacteria domain estimated by NGS. **(B)** Relative abundance (% of total ASVs) at genus level of ASVs belonging to the Bacteria domain estimated by NGS. Bacteria not identified were classified as ‘other genus’. *Ensifer* (Alpha-*Proteobacteria*), *Cupriavidus* (Beta-*Proteobacteria*), *Pseudomonas*, *Acinetobacter*, *Stenotrophomonas*, and *Pseudoxanthomonas* (Gamma-*Proteobacteria*).

The class- and genus-level classifications of the relative abundance of ASVs at 8 and 24 h are reported in Figures 4A,B, respectively. The prevalence of Gamma-*Proteobacteria* (>99%) was confirmed. The predominant genus was *Pseudomonas* with percentages of 88% at 8 h and 92% at 24 h, respectively. The *Pseudomonas* genus with *Acinetobacter*, *Stenotrophomonas* and *Pseudoxanthomonas* represented 99% at 8 h and 96% at 24 h of the overall bacteria identified. The sum of the percentages of the *Cupriavidus* genus belonging to Beta-*Proteobacteria* and *Ensifer* genus belonging to Alpha-*Proteobacteria* was just 1 and 4% at 8 and 24 h, respectively.

The predictive functional analysis showed 1866 genes in the bacterial consortium with no differences (*t* test non-significant) between the sampling times. Interestingly, 8 esterases, 49 ATP binding cassette (ABC) transporters and 3 genes involved in oxidative stress were found; the relative abundances are reported in Table 3.

**TABLE 3:** Relative abundance of gene families: esterase, oxidative stress and ABC transporters in the inoculum and at 8 and 24 h.

Esterase	Inoculum	8 h	24 h
Triacylglycerol Lipase	0.09%	0.12%	0.09%
Lysophospholipase	0.09%	0.11%	0.09%
Protein-glutamate Methylesterase	0.35%	0.39%	0.36%
Pimelyl-[acyl-carrier protein] Methyl Ester Esterase	0.09%	0.12%	0.10%
4-hydroxybenzoyl-CoA thioesterase	0.09%	0.10%	0.09%
Alkaline Phosphomonoesterase	0.01%	0.02%	0.01%
Glycerophosphodiester Phosphodiesterase	0.18%	0.17%	0.18%
Phosphoribosyl 1,2-cyclic Phosphate Phosphodiesterase	0.08%	0.08%	0.09%
**Sum of Esterases**	1.00%	1.10%	1.01%
Oxidative stress			
Glutathione peroxidase	0.28%	0.32%	0.28%
Glutathione-disulfide reductase	0.09%	0.09%	0.09%
Superoxide dismutase	0.18%	0.20%	0.19%
**Sum of ABC Transporters**	3.69%	3.37%	3.58%

## Discussion

The aim of the present work was to isolate and characterize a bacterial consortium capable of quickly degrading SLES for its potential seeding (bioaugmentation) on spoil material from EPB-TBM tunneling. The bacterial consortium, isolated at a real excavation site, was able to grow efficiently at SLES concentrations between 125 mg/L and 2 g/L in a few hours. It was able to degrade 94% of the anionic surfactant in only 9 h at an initial concentration of 250 mg/L, showing its high potential in removing SLES.

Our study is the first one, to our knowledge, which isolated a SLES degrading bacterial consortium from a deeper soil (10–20 m). The fact that a bacterial consortium and not a single species was isolated using SLES as the only carbon source is in line with what other authors found in wastewaters (Khleifat, 2006; Paulo et al., 2017; Fedeila et al., 2018). However, the bacterial consortia isolated in these works were able to degrade only 50% of SLES in 5 days at an initial concentration of 250 mg/L (Fedeila et al., 2018), 86% in 21 days at an initial concentration of 10 mg/L (Paulo et al., 2017) and 100% in 4–6 days at an initial concentration of 3 g/L (Khleifat, 2006), respectively. Recent studies report SLES as a biodegradable compound in foaming agent conditioned soil from tunnel excavation sites, with half lives of 6–9 days for initial SLES concentrations of 70–100 mg/kg (Barra Caracciolo et al., 2019). Moreover, detrimental effects (between 0 and 3 days) on bacterial numbers and cell viability were also observed, indicating that SLES is toxic for some bacterial groups inside the microbial community (Barra Caracciolo et al., 2019). Interestingly, in the same work an increase in Gamma-*Proteobacteria* was observed, as in our results, showing the ability of this bacterial group to resist the toxic effects of SLES and to degrade it. Surfactants can in fact affect the biological activity of microorganisms by binding to their enzymes, proteins or phospholipids, or by changing the hydrophobicity of their cells (Cserháti et al., 2002). Surfactants are known to be toxic for microorganisms in water solutions at critical micelle concentrations (CMC) and SLES is reported to form micelles at concentrations higher than 300 mg/L (Aoudia et al., 2009). Surfactant micelles can solubilize the membrane lipid of bacterial cells and lead to cell lysis (Glover et al., 1999; Li and Chen, 2009). The FISH analysis showed a predominance of Gamma-*Proteobacteria* and the NGS confirmed this result, showing that the bacterial consortium consisted mainly of bacterial genera (*Pseudomonas*, *Acinetobacter*, *Stenotrophomonas*, and *Pseudoxanthomonas*) belonging to this group. Gamma-*Proteobacteria* in fact possess esterase enzymes capable of breaking the SLES ester bond (Bornscheuer, 2002; Panda and Gowrishankar, 2005). The role in SLES degradation of the minor genera identified is not well known, although some authors found some Beta-*Proteobacteria* isolated from wastewater able to degrade SLES (Fedeila et al., 2018).

Finally, the functional profile analysis of the bacterial consortium selected confirmed the presence of the esterase gene family involved in SLES degradation. Moreover, the presence of genes associated to bacterial response to stress and contaminants (e.g., ATP-binding cassette transporters) would suggest that the bacterial consortium selected possesses defense mechanisms that helped it to counterbalance SLES potential detrimental effects (Davidson et al., 2008; Fajardo et al., 2019).

The bacterial consortium isolated can be potentially useful for routine bioaugmentation of spoil material where construction site constraints require a rapid decrease in SLES concentration. This can be the case of tunneling for a metro in a city or through mountains along a sea coast, where a prolonged storage is not possible because of lack of space and the spoil material would be forced to be considered a waste. The bioaugmentation of a bacterial consortium able to promote SLES degradation in a few hours can be an effective, low cost and nature-based solution.

## Conclusion

The present study reports the identification of a bacterial consortium capable of utilizing sodium lauryl ether sulfate as a sole carbon source and degrading it in a few hours. The identification of a natural bacterial consortium capable of degrading SLES has significant potential for bioaugmentation purposes, permitting a shortening of the storage time for soil excavated with foaming agents at construction sites and a safe promotion of the subsequent reutilization of the soil for different purposes. This work is an effective example of how low-cost nature-based solutions can be applied in real case studies while saving money in the carrying out of engineering works.

## Data Availability Statement

The datasets presented in this study are in an online repository. The name of the repository and accession number can be found at https://www.ebi.ac.uk/ena/browser/view/PRJEB37960.

## Author Contributions

All authors contributed to the article and approved the submitted version.

## Conflict of Interest

The authors declare that the research was conducted in the absence of any commercial or financial relationships that could be construed as a potential conflict of interest.

## Footnotes

1 https://qiime2.org

2 https://www.arb-silva.de/documentation/release-132/
